# Late Holocene echinoderm assemblages can serve as paleoenvironmental tracers in an Antarctic fjord

**DOI:** 10.1038/s41598-024-66151-5

**Published:** 2024-07-03

**Authors:** Giacomo Galli, Caterina Morigi, Ben Thuy, Karen Gariboldi

**Affiliations:** 1 Department of Environmental Sciences, Informatics and Statistics, University Ca’ Foscari Venice, Via Torino 155, 30172 Venice, Italy; 2 Department of Earth Sciences, University of Pisa, Via Santa Maria 53, 56126 Pisa, Italy; 3 Department of Paleontology, Natural History Museum Luxembourg, 25 rue Münster, 2160 Luxembourg, Luxembourg

**Keywords:** Climate sciences, Ecology, Environmental sciences, Ocean sciences, Solid Earth sciences

## Abstract

High Latitude fjords can serve as sediment trap, bearing different type of proxies, from geochemical to micropaleontological ones, making them exceptional tools for paleoenvironmental reconstruction. However, some unconventional proxies can be present and can be used to depict a comprehensive and exhaustive interpretation of past changes. Here, studying a sediment core in Edisto Inlet (Ross Sea, Antarctica) we used irregular echinoid spines and ophiuroids (*Ophionotus victoriae*) ossicles to trace environmental changes throughout the last 3.6 kyrs BP. Irregular echinoids can serve as proxy for the organic matter content, while *O. victoriae* ossicles can be used as proxy for steady sea-ice cycle along with organic deposition events. *O. victoriae* release a high number of ossicles, making estimation about the population quite challenging; still, presence data, can be easily collected. By applying Generative Additive Models to the stratigraphical distribution of these data, we detected an environmental phase that was previously unnoticed by other traditional proxies: the Ophiuroid Optimum (2–1.5 kyrs BP). In conclusion, here we demonstrate how echinoderm presence can be used as a valuable source of information, while proving the potential of modelling binary data to detect long-term trend in Holocene stratigraphical records.

## Introduction

Fjords are one of the most important transitional environments of the high latitudes. Recently they have been identified as carbon cycle hotspots making them an important factor in Earth’s climate system, being the marine environment with the highest carbon burial rate per unit area ^1,2^. However, most of the studied fjords are in the northern Hemisphere, especially in the Arctic, whilst the southern counterpart remains less studied with the Antarctic peninsula being the only exception^3^. In their role as sedimentation traps, fjords have been extensively used in paleoenvironmental reconstruction, especially to disentangle abrupt changes in the environment over the Holocene. This has been of great importance in the Antarctic Peninsula to recognize glacial advance or retreat, meltwater pulses, changes in the hydrographic conditions and in sedimentation regime^3–7^. A plethora of methods have been used to investigate these aspects: from micropaleontological analysis on foraminifera and diatoms^8^, to geochemical proxies and biomarkers^9^.

In addition, these highly dynamic systems have been studied for their modern ecosystem characteristic. Bae et al.^10^ showed that different species of diatoms, located in different parts of the Marian coves, can build up different macrofauna communities. Lagger et al.^11^, studying Potter Cove in 2010, found that ascidians and bryozoans constitute more than 90% of the total benthic community of a newly ice-free area.

Despite the increased attention towards the environmental evolution of fjords, little attention has been paid to the use of macrofauna component in paleoenvironmental reconstruction regardless of their importance as a tool to give insights on community and environmental dynamics^12,13^.

Whitin this context, our study focuses on echinoderm remains in the Edisto Inlet, a small fjord located in the Ross Sea characterized by a seasonal sea-ice cover to assess if macrofaunal component can be used to depict a more comprehensive view of the environmental evolution of this area. The fjord has been previously studied for paleoenvironmental dynamics, seafloor characteristics and tephra content^14–18^. The bottom of the Inlet is covered by a 110 m thick expanded laminated Holocene sedimentary sequence, reflecting different diatom communities through distinct lamina colours^17,18^. Although echinoderm remains (ophiuroids and echinoids) were previously used to develop the age-depth model of two cores inside the Inlet, no analysis on their stratigraphical distribution was conducted^18,19^.

Echinoderms are one of the most important benthic groups in Antarctic waters, constituting most of the total benthic biomass^20–22^. Moreover, this group plays a fundamental role in the community structure making it a valuable source of information for ecosystem dynamics^23–26^. Upon decay, echinoderms release a high number of ossicles (several thousand per individual in case of the ophiuroids). Thanks to recent advances in understanding of lateral arm plate (LAP) morphology of ophiuroids, aligned with genomic studies, isolated ophiuroid ossicles have been identified as powerful tool for micropaleontological analyses^27–29^. The goal of this study is to provide a case study for the use of echinoderm ossicles as an ecological and paleoenvironmental proxy and thereby unlock the micropaleontological potential of echinoderms as key macrofaunal component.

### Study area

**Figure 1:**
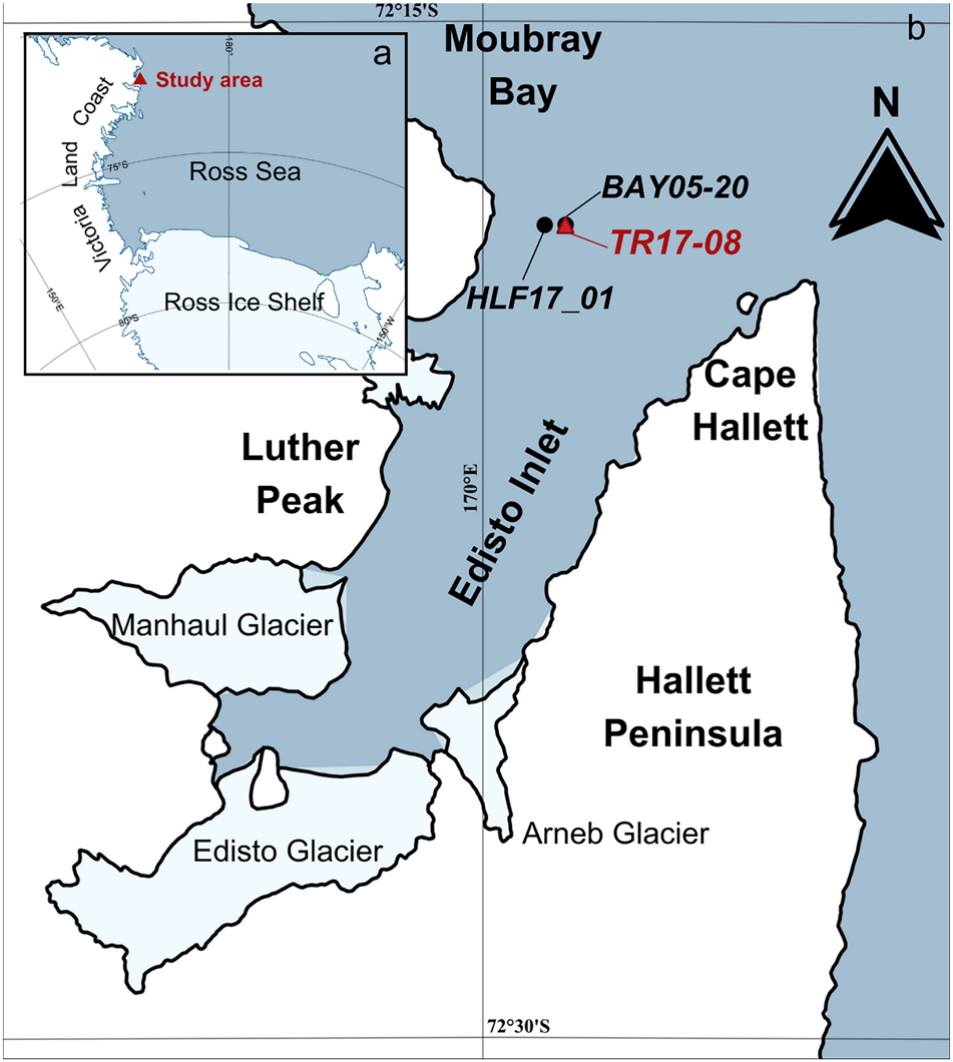
(**a**) Ross Sea and the studied area; (**b**) marine sediment cores location in Edisto Inlet. The red triangle highlights the core used in the study. Black points indicate marine cores retrieved from the area and used for comparison: HLF17-01^18^ and BAY05-20^67^. Map from Galli et al.^15^.

Edisto Inlet, situated in the northwestern part of the Ross Sea, is an elongated and narrow fjord with an average depth of 500 m and a minimum depth of 100 m at the fjord mouth^14,17^ (Fig. 1). The fjord has four different glacial inputs: the Edisto glacier, the Manhaul glacier, and the Arneb glacier and a little unnamed glacier near the mouth(Fig. 1). Geomorphological evidence, combined with geochemical and micropaleontological analyses, was used previously to study the environmental evolution of the Inlet over the last 11 kyrs BP^15,17,18^.

From 11 kyrs BP, the glaciers started to retreat, leading to open water conditions, along with the reinvigoration of the circulation. The period from 9 to 2.6 kyrs BP witnessed a deglaciation phase with the establishment of a seasonal sea-ice regime^17^. Subsequently, from 2.6 to 0.7 kyrs BP, geochemical and foraminifera analysis suggested the presence of a seasonal sea-ice cover, and around 1.48 kyrs BP, conspicuous glacial meltwater flow attributed to the retreat of three in-situ glaciers was linked with the onset of the Medieval Climate Anomaly (MCA)^15,18^. Lastly, spanning from 0.7 kyrs BP to the present day, after the onset of the Little Ice Age (LIA), a prolonged period of sea-ice cover has been suggested by the sudden decrease of the sedimentation rate by one order of magnitude, probably due to persisting presence of the sea-ice during the thawing season^15,16,18^.

## Materials and methods

### Core TR17-08

The marine sediment core TR17-08 (14.6 m long) was retrieved in January 2017 at the entrance of the fjord at a depth of 462 m below sea level (Fig. 1). The core consists of diatomaceous ooze and shows a lamination defined by the alternation of olive-green to brownish lamina (dark), and white ones (light). In Edisto, lamina colour reflects diatom assemblages: dark laminae are dominated by *Fragilariopsis curta, F. obliquecostata* and have a low biovolume content of *Corethoron pennatum*, indicative of a first sea-ice break-up during the early austral summer, whilst light laminae are mainly composed by *C. pennatum* and are deposited during the later part of the summer, when oligotrophic conditions are present^18^ (Fig. S1). The age depth-model was constructed using 10 radiocarbon dates and 1 tephra layer associated with the Mount Rittmann eruption^15,16^ (Fig. S2). The core covers a period of 3.6 kyrs BP and, around 0.7 kyrs BP, in concomitance with the Mount Rittman tephra, the sedimentation rate changes from an average of 0.47 cm yr^−1^ to an average of 0.07 cm yr^−1^^16,18^ (Fig. S2).

### Micropaleontological samples

Samples with 1 cm thickness were taken every 10 cm from core TR17-08, resulting in a total of 152 samples. Samples were washed with a 63 µm sieve and dried overnight at 40 °C. Ice Rafted Debris (IRD) content was determined by counting sharp clastic remains in the > 1 mm fraction. Foraminifera and echinoderm remains in the > 150 µm fraction were picked exhaustively. Since autotomy and arm regeneration can exaggerate the number of ossicles released into the environment by ophiuroid individuals^30^, we used the distribution of ossicles to evaluate the presence/absence of the echinoderm taxa. Benthic Foraminifera Accumulation Rate (BFAR, specimens cm^−2^ yr^−1^), Planktic Foraminifera Accumulation Rate (PFAR, specimens cm^−2^ yr^−1^) and IRD fluxes (counts·cm^−2^ yr^−1^) were measured as described in Herguera and Berger^31^. Foraminifera were recognised at the species level (for an extensive list of the species see table S1) using the taxonomic table from^32–36^. Ophiuroid remains were analysed using Scanning Electron Microscopy (SEM) to evaluate the diagnostic features of the LAPs^29,37^. Echinoid spines were collected and recognised at the functional level.

### Statistical analysis

Presence/Absence data are difficult to interpret due to their binomial nature. Generative Additive Models (GAM) were used to model the probability of occurrence over time, by utilizing the method to detect trends in temporal series described by Simpson^38^. GAM are homologous to the LOESS curve but rely on fewer assumptions, giving these models an advantage since they can detect significant trends in temporal series ^38,39^. The age (in yrs BP) of the layer was used as a predictor of the model. GAM were applied to both echinoid and ophiuroid distribution. Statistical analyses were performed in the software RStudio^40^ (v4.3.1). GAM were calculated using the *mgcv* package^41^. The fit criterion used was the restricted maximum likelihood (REML, Simpson, 2018). For the stratigraphical analysis, a stratigraphically constrained analysis with Euclidean distance (CONISS) was computed using BFAR, PFAR, IRD, Margalef (M), Eveness (J) and the probability of occurrence of the echinoids (P(E)) and ophiuroids (P(O)). We used the package *tidypaleo* for the CONISS, which uses a broken stick approach to define significant clusters^42^. Foraminifera diversity indexes, M and J, were calculated using the software PAST^43^ (v4.14). M index is calculated using the formula $M = \left( {S - 1} \right)/ln(n$), where S is the number of species and n is the total number of individuals. J is calculated using the formula $J = e^{H} /S$, where H is the Shannon Index, corresponding to $H = - \mathop \sum \limits_{i} \frac{{n_{i} }}{n}\ln \frac{{n_{i} }}{n}$.

## Results and discussion

### Ophiuroids and echinoids

**Figure 2:**
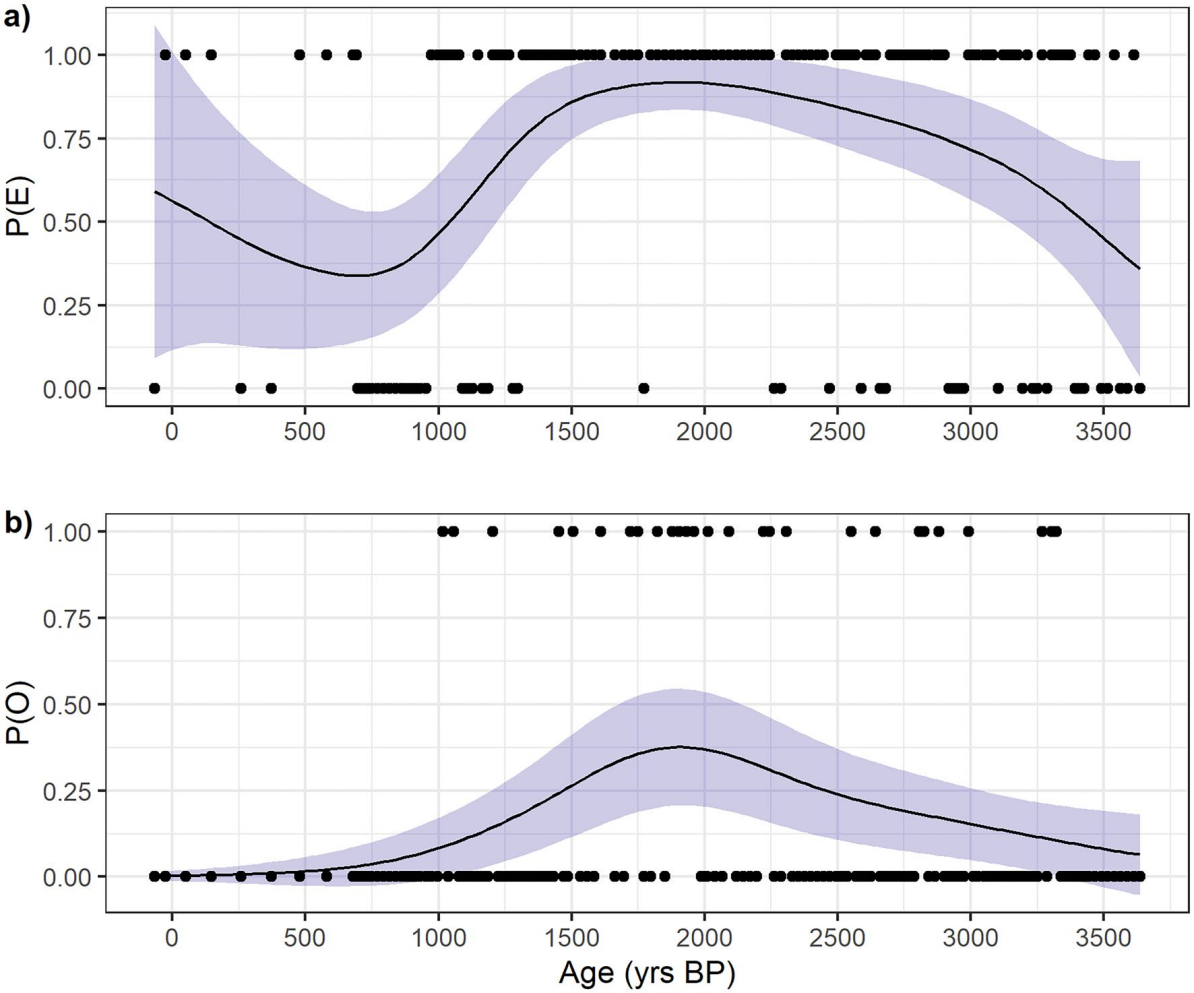
GAM model of the echinoderm’s distribution. (**a**) GAM model of the irregular echinoids; (**b**) GAM model of the ophiuroid*.* P(E) refers to the probability of occurrence of the echinoids, while P(O) is the probability of occurrence of the ophiuroids. Trend line is displayed as black line, and the confidence band (95%) is indicated by the light blue ribbon. Black points represent the presence (1)/ absence (0) distribution along the core.

In our record echinoids were more common throughout (104/152 samples, 69%) than ophiuroids (27/152 samples, 18%) but were consistently absent from 1 to 0.7 kyrs BP (Fig. 2). Ophiuroid presence is more frequent during the period that goes from the 3.6 to 1 kyrs BP. GAM applied to the distribution shows significant trends (p < 0.05), increasing their values as the density of the presence points increases (Fig. 2; Table S2). The P(E) increases from 3.6 to 1.5 kyrs BP, while it tends to decrease after 1.5 kyrs, where the echinoderms are absent (Fig. 2a). Ophiuroids show a different trend: the P(O) increases steadily from the bottom and peaks around 2 kyrs BP, in concomitance with the interval with most presence points (Fig. 2b). Although the peak of the P(O) is low (< 0.5), we argue that these low values arise due to the low number of presence points of the ophiuroid distribution (Fig. 2b). Despite the low probability values, the P(O) still gives important clues on the long-term trend, as it can define the frequent presence of ophiuroids, as seen by the bulging of the curve in the interval 2–1.5 kyrs BP (Fig. 2b).

This application shows the strength of utilizing GAM models even in binomial distribution, unlocking possibilities on the use of this type of models to understand long-term macrofauna dynamics just by a presence/absence matrix derived from a temporal series.

**Figure 3:**
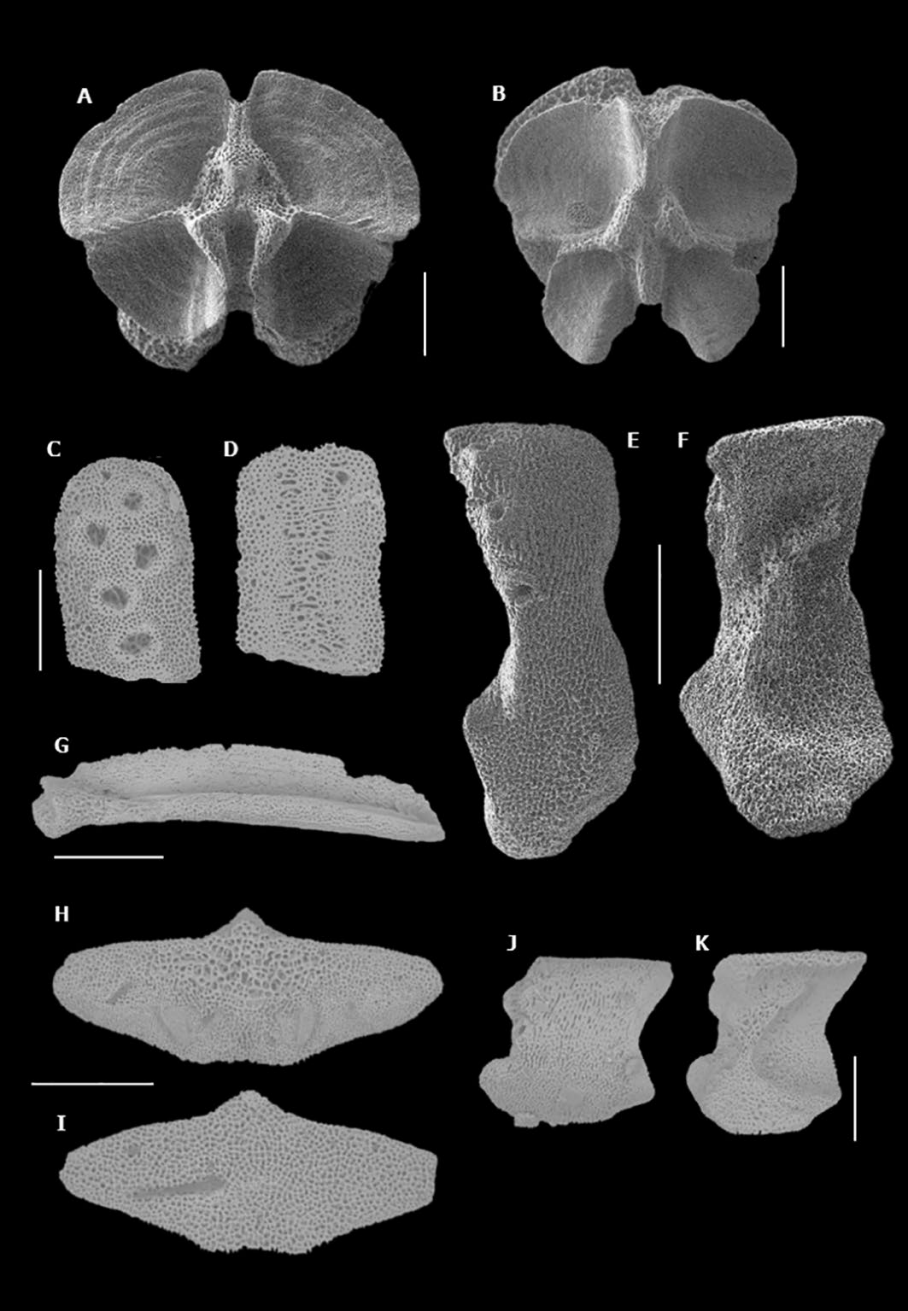
Skeletal plates of *Ophionotus victoriae* (scale bars = 500 µm): (**A**, **B**) vertebrae, proximal and distal views; (**C**, **D**) broken Dental plates, external and internal view; (**E**, **F**) proximal lateral arm plate, external and internal views; (**G**) adradial genital plate; H-I. Dorsal arm plate, internal and external views; (**J**, **K**) distal lateral arm plate, external and internal views.

**Figure 4:**
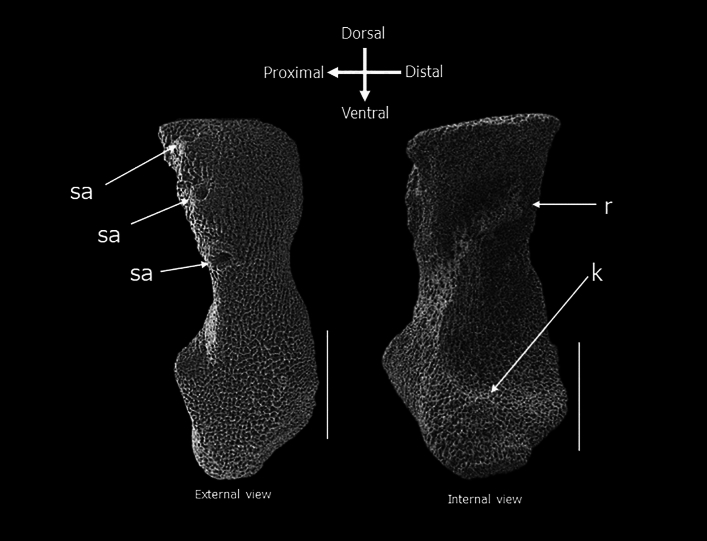
Lateral arm plates of *Ophionotus victoriae*. sa, spine articulation; r, ridge; k, knob. Scale bar = 500 µm.

Micromorphological analysis of the LAPs retrieved from our samples allowed us to recognize the presence of the species *Ophionotus victoriae* Bell, 1902^29,37,44^. Since there were no other types of LAP throughout the core, we assume that all other ophiuroid ossicles belonged to the same species (Figs. 3 and 4).

*Ophionotus victoriae* is a well-known and widely distributed species across Antarctica, living at almost every depth and within different types of environments^20^. Like other ophiuroids in Antarctica, *O. victoriae* is an opportunistic species with a high diet plasticity, even showing cannibalistic behaviour in high-density populations ^45^. Cannibalistic behaviour is typically found among organisms that live in areas affected by strong seasonal fluctuations, like fjords or high-latitude enclosed basins, generally characterized by a seasonal cycle of the sea-ice cover^26,45,46^. In Deception Island, *O. victoriae* spatial distribution relates to the sedimentation regime and a low ice-related disturbance^21,47^. In addition, *O. victoriae* has a peculiar reproductive biology involving synchronous annual spawning in November–December, long oocyte development periods and, the dependence upon the previous year sedimentation event of the reproductive effort^48^. Considering these premises, we hypothesize that the presence of *O. victoriae* could be a valuable proxy for low interannual variability of the environmental cycle of the study area that corresponds to a seasonal sea-ice cycle along with constant organic deposition events. Given the crucial role of *O. victoriae* in the bentho-pelagic coupling of the community, its presence could also be used as a proxy to indicate a mature benthic community, implying that an energy flux was present from the primary producers (e.g. diatoms) to the secondary consumers^24^.

Since the skeleton of *O. victoriae* is made of high-Mg calcite, the ossicles can be prone to dissolution after decay, with a complete disappearance estimated to occur within 6–105 years^49^. However, in high sedimentation regimes, like the Edisto Inlet, rapid burial could have removed the ossicles from corrosive water masses enabling their preservation^50^. Considering the similar chemical composition of *O. victoriae* ossicles and echinoid spines and considering the coupled presence of both along the core, we infer that there was no taphonomical filter on the distribution of the echinoderm remains^51^.

Echinoid spines were identified as belonging to irregular echinoids (infraclass Irregularia, Latreille 1825, Fig. S3) due to their morphology, although the material available precluded a species-level identification^52,53^. Irregular echinoids are bottom dwellers feeding on organic matter on and within the seafloor^54^. This type of ecology hints at a possible use as proxy for organic matter content on the seafloor.

### Paleoenvironmental reconstruction over the Late Holocene of Edisto Inlet

**Figure 5:**
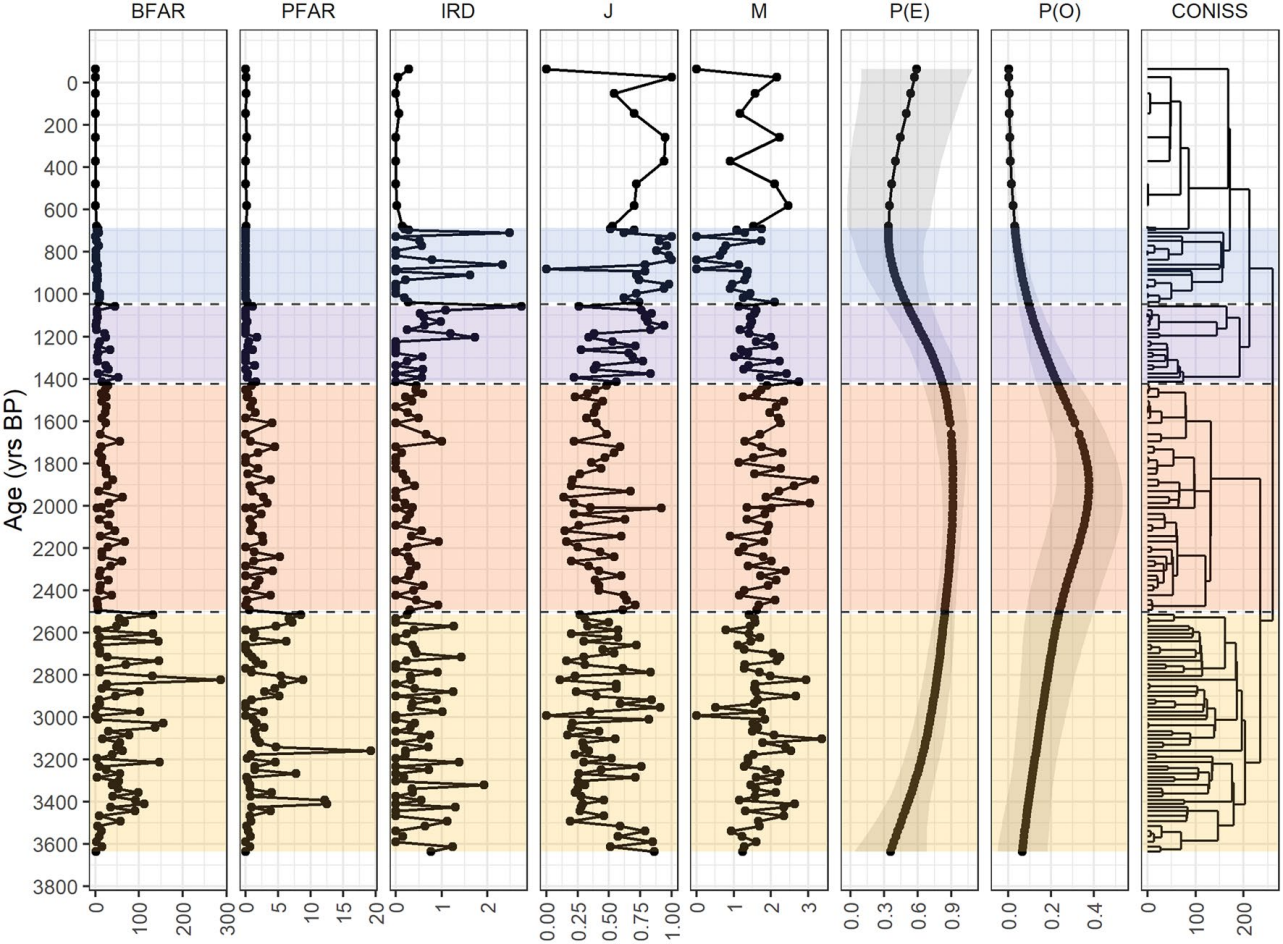
Comparison between the BFAR, PFAR, IRD, J, D, P(O) and P(E) over 3.6 kyrs BP from core TR17-08. CONISS cladogram is also displayed. The dashed lines represent the CONISS cluster division. Note the different scale on every graph. BFAR, PFAR and IRD are calculated in counts·cm^−2^·yr^−1^. The grey ribbon in the P(O) and P(E) graph represents the 95% confidence interval.

**Figure 6:**
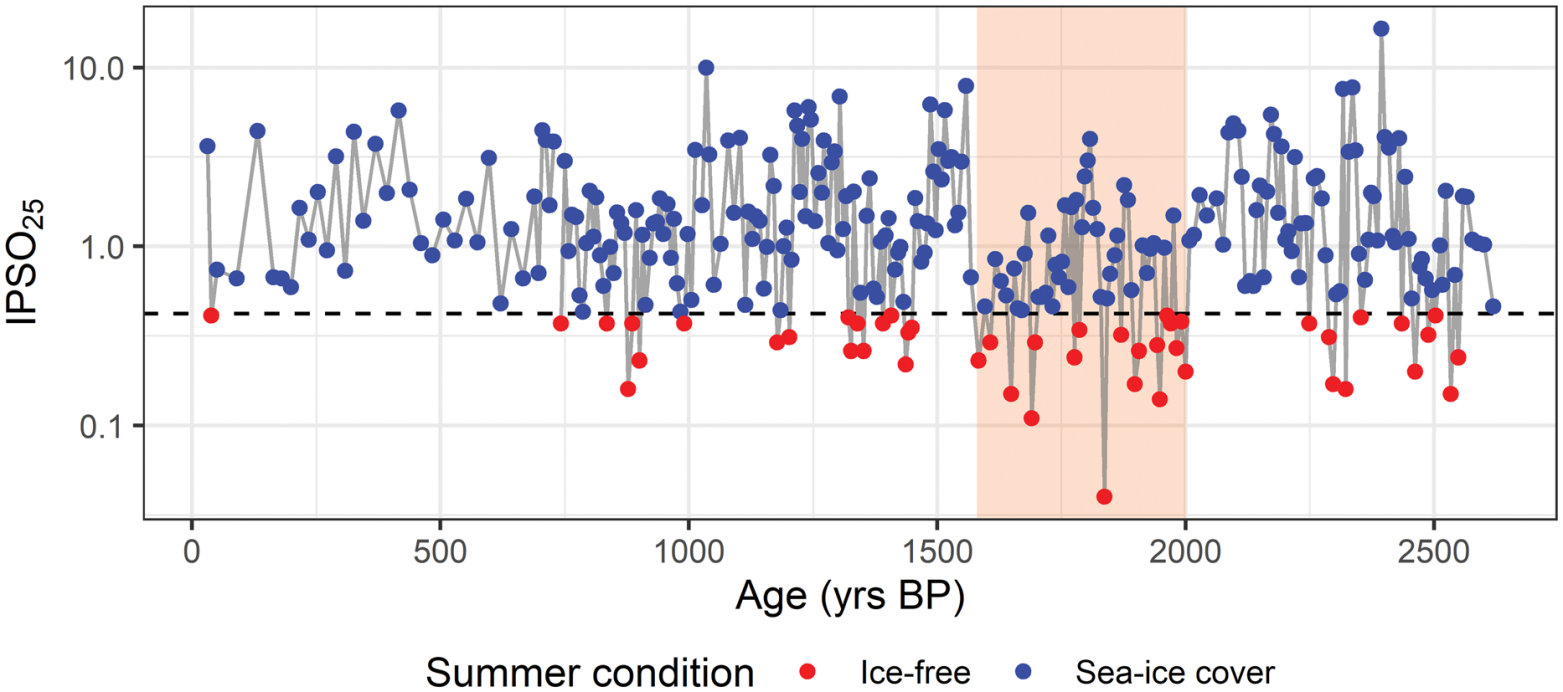
IPSO_25_ values over the last 2.6 kyrs BP in the core HLF17-01 (Tesi et al.^18^). The blue dots indicate summer sea-ice cover while red dots indicate free-sea ice conditions during the summer, the red shaded area highlights the period where P(E) and P(O) reaches the maximum. The y-axis is in logarithm scale.

To test whether the echinoderms could be used as a proxy for past environmental conditions, we compared P(E) and P(O) with other proxies derived from the core TR17-08 and from biogeochemical proxies derived from a nearby core inside the Inlet, HLF17-01 (Figs. 5 and 6). BFAR and PFAR have been used extensively as proxy for the paleo productivity^31,55^. Also, IRD fluxes have been used to reconstruct the location of the polar front, iceberg discharging events and the extension of the sea-ice cover at both poles^56–59^. In Barilari Bay (Antarctic Peninsula), a fjord with similar environmental features as Edisto, high abundances of IRD suggests the onset of a discharging event guided by calving of an iceberg in a marine terminating glacier, and a decrease in its content marks the onset of seasonally open marine conditions^59^.

Changes in J and M have been related to change in the benthic environment as well as the presence of stressful periods or events^60–62^. In the Arctic, changes in the population of calcareous foraminiferal fauna diversity and densities are related to changes in the macrofauna component, paralleling the response of the macrofauna diversity^62^. In our study, the diversity indexes calculated on the foraminiferal content are used as a proxy for the patchiness of the environmental conditions. Foraminiferal fauna composition depends upon the physical and chemical characteristics of the environment. Thus, an increase in the number of species could be evidence of an increase in the number of habitats or ecological niches^61^. On the other hand, J is used to evaluate how well the species are partitioned in a community, with lower values indicating that few species are dominating the community^63^. This can be interpreted as the prominent presence of the ecological characteristics of the dominant group. In other words, if J decreases and M increases, we can suppose that the decrease in the equitability (increase in the dominance of a species or a group) and the contemporary increase in the number of species, reflects the prominent presence of an environment type (indicated by the low values of J), while new rare species are added (indicated by M). In turn, this implies the steadiness of the ecological condition, since one species (or group) is dominating the assemblages, while not taking into consideration the environmental condition itself.

Considering these premises, the values of P(O) and P(E) should be higher in concomitance with relatively high primary productivity (High BFAR and PFAR), seasonal sea-ice cycle (low content of IRD), and an increase in the environmental steadiness (low J, high M).

The CONISS analysis divided our record into 4 clusters (Fig. 5, dashed lines). Starting from the oldest, the first cluster (Fig. 5, yellow band) goes from 3.6 to 2.5 kyrs BP. Within this interval the BFAR and PFAR show similar patterns, indicating a period with explosive increases of the primary productivity^55^. IRD fluxes are relatively high with respect to the subsequent zone (Fig. 5, red band), while J and M do not show any trend. This can be interpreted as a climatic phase where primary productivity was high but inconsistent from year to year, indicated by the peaks of IRD (Fig. 5, yellow band). The same conclusion can be inferred by the peaks in the BFAR and PFAR content and by the variability of J^59,64^ (Fig. 5, yellow band). The increase in the P(E) and P(O) corroborates this view. The increase in P(E) suggests an increase in the organic matter content on the seafloor probably derived from superficial algal blooms along with the increase in P(O) that can be interpreted as a gradual increase in the stability of the seasonal sea-ice cycle.

The subsequent interval (2.5–1.4 kyrs BP, Fig. 5 red band) is characterized by low values of BFAR, PFAR and IRD. During this time, J decreases in the middle part along with an increase in M. The low values of BFAR and PFAR can be interpreted as a decrease in primary productivity alongside a decrease in the variability of the input of nutrients, indicated by the lower values of the peaks (Fig. 5, red band). The IRD content is lower than the previous interval (Fig. 5, yellow band) and shows less variability, indicating the presence of a seasonal sea-ice cover^59^. During this period, P(O) and P(E) peaks at around 2–1.8 kyrs BP, validating our hypothesis that ophiuroid presence/absence can be used as a paleoenvironmental proxy, since the premises stated previously are met: low IRD content, relatively high BFAR and PFAR, and a decrease in J along with an increase in M.

In addition, this interval can be compared with the geochemical data from core HLF17-01 and with the foraminifera analysis from core TR17-08^15,18^. In the study of Tesi et al.^18^, IPSO_25_ index (Ice Proxy for the Southern Ocean with 25 highly branched isoprenoids^65^) have been used to track absence of sea-ice cover during the austral summer, revealing a prominent ice-free summer season from 2.6 to 0.7 kyrs BP (Fig. 6). However, the interval with the average lower values and with the most frequent under-the-threshold values of the IPSO_25_ spans the interval from 2 to 1.5 kyrs BP (Fig. 6). This period also corresponds to the peak in both P(E) and P(O) values, corroborating the hypothesis on the use of *Ophionotus victoriae* as a proxy for seasonal sea-ice cycle (Fig. 6). Foraminiferal analysis also supported the view of a prominent seasonal phase during this period^15^. By combining the results from the geochemical and the foraminiferal analyses with our echinoderm distribution we can identify a time interval that is characterized by an interannually stable sea-ice cycle with conspicuous organic sedimentation events, along with an energy flux that goes from primary producers (diatoms) to secondary consumers (ophiuroids and echinoids), implying the presence of a developed benthic community. We call this period Ophiuroid Optimum due to the relationship between the ophiuroid presence and the IPSO_25_ value (Figs. 5 and 6).

From 1.4 to 0.7 kyrs BP a transitional phase took place (Fig. 5, purple and blue bands). BFAR and PFAR drop to near 0 values, while IRD content suddenly increases at 1.2 kyrs BP. J increases, while M decreases in concomitance with P(E) and P(O) over the same period (Fig. 5, purple band). The period after 0.7 kyrs BP (Fig. 5, white band), experiences a drop in the sedimentation rate from an average value of 0.49 to 0.07 cm yr^−1^ and we decide to divide it from the previous cluster manually, since it yields less resolution, suggesting a closed environment with a sea-ice cover that does not thaw during the summer, or experience very incipient opening^15,18,19^. Foraminiferal analysis over 1.4–0.7 kyrs BP suggests the presence of a conspicuous meltwater flow, probably derived from the retreat of in-situ glaciers with the onset of the MCA, a warm phase recognised in the Northern Hemisphere as well as in the Antarctic Peninsula and in the Victoria Land Coast ^15,66^. The transitional phase has been interpreted as a deterioration of the seasonal sea-ice cycle, with an increase in the residence time of water masses inside the fjord^15^. The increase in J in concomitance with a decrease in M of the foraminiferal community also suggests a transitional state, where species vanish from the community, while the remaining ones thrive.

In addition, from another nearby core, BAY-0520 (Fig. 1), the increase in the *Fragilariopsis curta* content, a sea-ice indicator diatom, over the same period, is coherent with our interpretation^67^. Thus, our results suggest a period of less stable seasonality of the sea-ice cover, with a prolonged season of the winter cover, as suggested by the low BFAR and PFAR values. IRDs have the highest values over this period, and the steady decreases of P(E) and P(O), along with the increase in J and the decrease in M, corroborate the hypothesis of a reduction in the seasonality as well as the decrease in productivity, culminating around 0.7 kyrs BP^15,18^. In addition, the absence of both echinoids and ophiuroids (Fig. 2) suggests the absence of an energy flux that goes from the primary producers to the secondary consumers, reflected in the absence of a mature benthic macrofaunal community.

The period from 0.7 kyrs BP to recent (Fig. 5, white band) has been associated with the onset of the LIA, a northern hemisphere cooling period that has also been identified in Antarctica ice cores by a sudden decrease of 2 °C in the reconstructed air temperature^68,69^. Over this period, the sedimentation rate in Edisto Inlet is stable but with low value, suggesting the presence of a prolonged period of sea-ice cover with low-to-no productivity^15,18^. Results from P(E) and P(O) are difficult to interpret due to the low number of samples with respect to the previous zones, and the increase in their value and in the amplitude of the confidence interval could be derived from the low resolution of this period compared to other ones (Fig. 5).

## Conclusion

In this study we evaluated the use of microfossils of macrofaunal organisms as paleoenvironmental proxies in high sedimentation settings by studying the marine sediment core TR17-08 in the Edisto Inlet, Ross Sea (Antarctica). In the record, spanning over 3.6 kyrs BP, we were able to successfully identify the presence of an ophiuroid species, *Ophionotus victoriae,* by analysing lateral arm plate (LAP) morphology along with the presence of irregular echinoid spines. To detect significant trends in the stratigraphical distribution of the latter, a presence/absence matrix was constructed. Generative Additive Models (GAM) were used to convert a binomial distribution into a continuous distribution, serving as a new way of analysing macrofaunal presence in paleoenvironmental studies. By comparing presence of echinoids (P(E)) and ophiuroids (P(O)), respectively, with other biogeochemical and micropaleontological proxies, we were able to demonstrate the use of *O. victoriae* as a proxy for the interannual steadiness of the seasonal sea-ice cycle and the organic sedimentation events, as well as a functional bentho-pelagic coupling. Although the irregular echinoid spines could not be identified in detail, their presence can be used to infer the existence of organic matter at the seafloor.

By utilizing geochemical, micropaleontological and macrofaunal proxies we were able to identify four different phases, in accordance with previous studies and a new seasonally stable phase called “Ophiuroid Optimum”^15,18^.

This study demonstrates that macrofaunal components can be used in micropaleontological studies and in multiproxy approaches to reconstruct paleoenvironmental settings, opening new ways to describe and interpret past climatic and environmental changes.

### Supplementary Information

Supplementary Information 1.

Supplementary Information 2.

## Data Availability

The data used to produce the results in this study are available in the supplementary material of the article.
