# Supplementary material for: Late Holocene echinoderm assemblages can serve as paleoenvironmental tracers in an Antarctic fjord
Source: Sci Rep. 2024 Jul 3;14:15300. doi: 10.1038/s41598-024-66151-5 (PMC11222549; doi:10.1038/s41598-024-66151-5)
Supplement: Supplementary file 1 — Supplementary Information 1. [file 41598_2024_66151_MOESM1_ESM.pdf]

Fjords, Echinoderms and the Holocene: a new ecological proxy from the Ross Sea (Edisto Inlet) – Supplementary information

Giacomo Galli[1,2], Caterina Morigi[2], Ben Thuy[3] and Karen Gariboldi[2]

[1] Department of Environmental Sciences, Informatics and Statistics, University Ca' Foscari Venice, Via Torino 155, 30172, Venice, Italy

[2]Department of Earth Sciences, University of Pisa, Via Santa Maria 53, 56126, Pisa, Italy

[3]Department of Paleontology, Natural History Museum Luxembourg, 25 rue Munster, L-2160 Luxembourg, Luxembourg

Core TR17-08

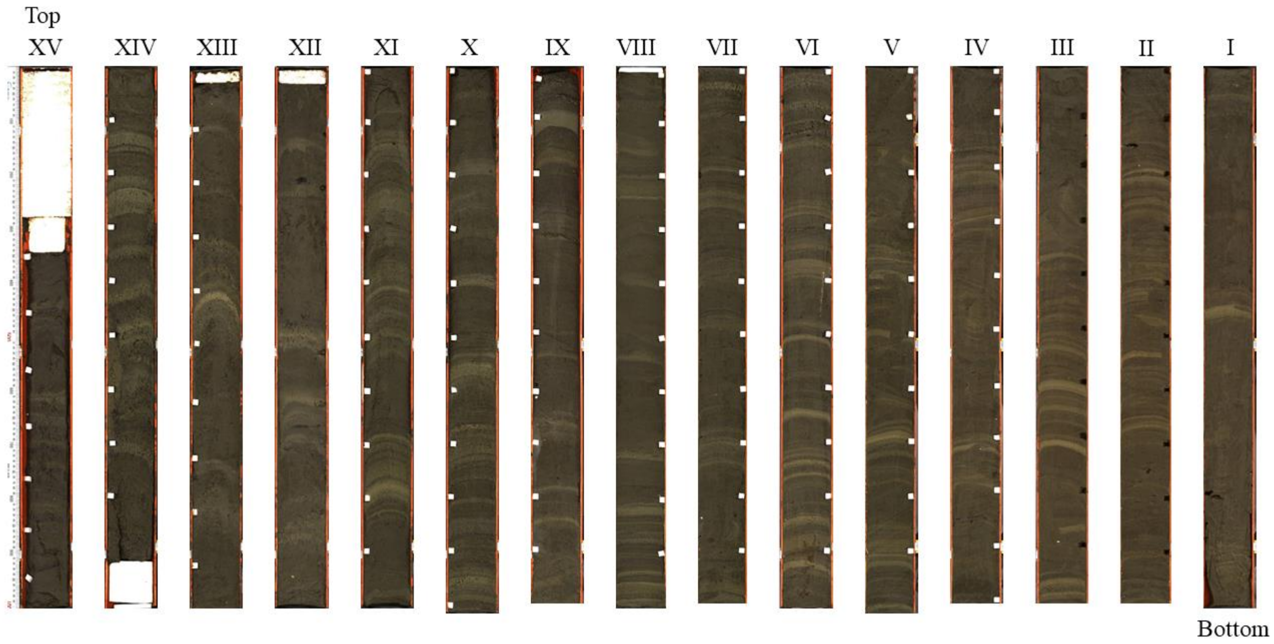

Figure S1. The 15 sections (ca. 1 m long) of the core TR17-08. Laminations (light and dark laminae) are present throughout the core. Notice the variability of the lamination sequences and the thickness of the laminae.

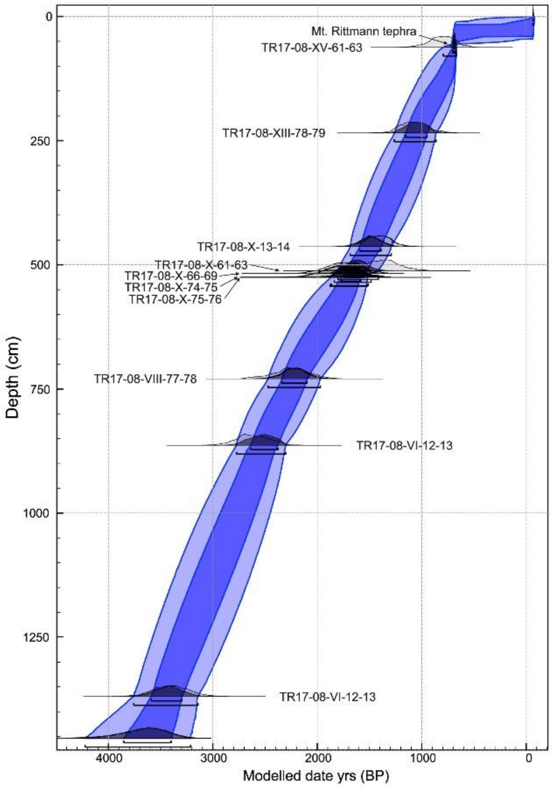

Figure S2. Age-depth model of the core TR17-08. The light blue band corresponds to 95% confidence interval, whilst the blue corresponds to the 65%. Distribution of the age and the name of the radiocarbon samples are displayed. Notice the abrupt change in sedimentation rate around 700 yrs BP (Di Roberto et al., 2023).

Foraminifera species

Test	Species	
**Agglutinated**	*Labrospira jeffreysii*	*Portatrochammina bipolaris*
	Paratrochammina bartrami	*Pseudotrochammina arenacea*
	Paratrochammina tricamerata	*Textularia earlandii*
	Trochammina nana	*Trochammina multiloculata*
	Portatrochammina antarctica	*Rhumberella* sp.
**Calcareous imperforate**	*Miliammina arenacea*	*Quinqueloculina seminulum*
	Miliammina oblonga	*Quinqueloculina venusta*
	Pyrgo depressa	*Quinqueloculina weaveri*
	Pyrgo williamsoni	*Quinqueloculina* spp.
**Calcareous perforate**	*Ammodiscus sp.*	*Lagena sp.*
	Astrononion antarcticus	*Melonis barleeanum*
	Astrononion echolsi	*Nonionella iridea*
	Cassidulinoides porrectus	*Nonionella bradii*
	Cibicides lobatulus	*Nonionella magnalingua*
	Fissurina semimarginata	*Nonionella sp.*
	Fissurina spp.	*Oolina hexagona*
		Patellina corrugata

	Globocassidulina biora	*Procelarogena multilatera*
	Globocassidulina crassa	*Pullenia quinqueloba*
	Globocassidulina subglobosa	*Rosalina globularis*
	Globocassidulina spp.	*Rosalina villaderboana*
	Heronallenia kempii	*Stainforthia feylingi*
	Hyalinonetrion sahulense	*Stainforthia* sp.
		Trifarina angulosa

Table S1. Lists of the foraminifera species identified divided by their test characteristics.

Irregular Echinoid spines

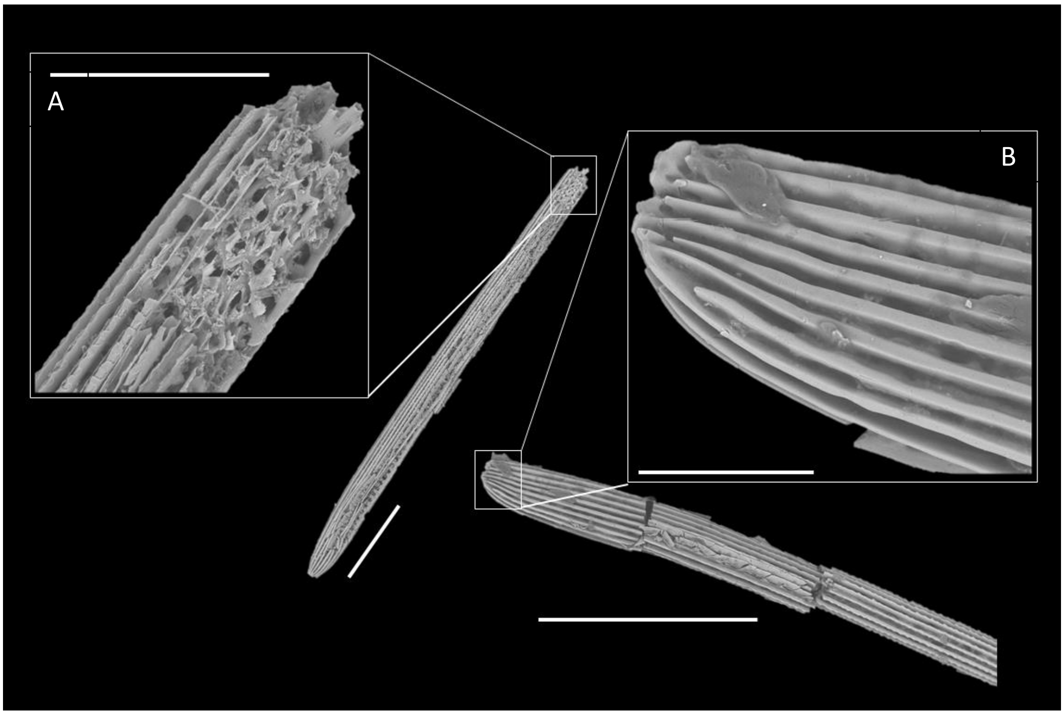

Figure S3. Examples of irregular echinoids spine from the core TR17-08. Photo taken with the SEM. A) Magnification of the basal part of the spine; B) Magnification of the basal part of the spine. Scale bar = 500 µm

Generative additive model results (GAM)

We report the GAM results from the package *mgcv* (Wood Simon, 2001). The smoothing algorithm selected for all the GAMs was the REML (Simpson, 2018). P(E) refers to the echinoids model, while P(O) refers to the ophiuroids model. The k-index of the *gam.check.* function describes if the number of knots chosen are enough to approximate the data. if the k-index value is too far from 1, then the number of knots should be increased because there is not enough basis to construct the model (Simpson, 2018).

GAM	Number of knots	Deviance explained	k-index	p- value parametric coefficients	p-value of the smooth terms
P(E)	15	17.3%	0.71	$p < 0.0001$	$p < 0.001$
P(O)	15	11.6%	0.97	$p < 0.0001$	$p < 0.05$

Table S2. Generative additive models result for the echinoid distribution, P(E), and for the ophiuroid distribution P(O).
